# Physiological and Metabolic Effects of *Opuntia ficus indica* spp. Peel Formulations

**DOI:** 10.3390/life15020148

**Published:** 2025-01-22

**Authors:** José Arias-Rico, Iris Cristal Hernández-Ortega, Osmar Antonio Jaramillo-Morales, Nelly del Socorro Cruz-Cansino, Quinatzin Yadira Zafra-Rojas, Olga Rocío Flores-Chávez, Rosa María Baltazar-Téllez, Esther Ramírez-Moreno

**Affiliations:** 1 Área Académica de Enfermería, Centro de Investigación Interdisciplinario, Instituto de Ciencias de la Salud, Universidad Autónoma del Estado de Hidalgo, Circuito Ex Hacienda, La Concepción S/N, Carretera Pachuca Actopan, San Agustín Tlaxiaca 42160, Hidalgo, Mexico; jose_arias@uaeh.edu.mx (J.A.-R.); ofloresc@uaeh.edu.mx (O.R.F.-C.); rosa_baltazar@uaeh.edu.mx (R.M.B.-T.); 2 Área Académica de Nutrición, Centro de Investigación Interdisciplinario, Instituto de Ciencias de la Salud, Universidad Autónoma del Estado de Hidalgo, Circuito Ex Hacienda, La Concepción S/N, Carretera Pachuca Actopan, San Agustín Tlaxiaca 42160, Hidalgo, Mexico; cristal.hernandez@cecyteh.edu.mx (I.C.H.-O.); ncruz@uaeh.edu.mx (N.d.S.C.-C.); quinatzin_zafra@uaeh.edu.mx (Q.Y.Z.-R.); 3 División de Ciencias de la Vida, Departamento de Enfermería y Obstetricia, Campus Irapuato-Salamanca, Universidad de Guanajuato, Ex Hacienda el Copal, km. 9 Carretera Irapuato-Silao, AP. 311, Irapuato 36500, Guanajuato, Mexico; oa.jaramillo@ugto.mx

**Keywords:** dietary fiber, cactus pear peel, intestinal transit

## Abstract

The objective of this study is to determine the physiological and metabolic effects of administration of dietary fiber formulations to male Wistar rats. The study population was divided into five groups to which food and water were orally administered ad libitum (control), alongside *Psyllium plantago*, sennosides A and B, cactus pear peel powder, and cactus pear peel tablet powder for 28 days. Body weight, biochemical parameters, fecal moisture, and intestinal transit were determined. The administration of the fiber formulations did not cause differences between the groups and they maintained a healthy weight; however, the consumption of the cactus pear peel tablet powder decreased serum glucose (127.85 ± 5.37 to 68.30 ± 12.48 mg/dL) in rats in a similar form to *Psyllium plantago* (127.85 ± 5.37 to 96.96 ± 3.26 mg/dL) in comparison with commercial products for rats, and the cactus pear peel powder had lower triglyceride levels (49.52 to 74.44 mg/dL) than commercial products at the end of the treatment. The samples maintained normal HDL levels with the exception of *Psyllium plantago* that had a decrease in treatment after 28 days. The administration of formulations of dietary fiber of cactus pear peel had physiological and metabolic effects similar to those of commercial products without change in the growth of the animals. Therefore, it could be used in the pharmaceutical or food industry.

## 1. Introduction

The cactus pear (*Opuntia ficus-indica* spp.) is a fruit belonging to the family of cacti of the *Opuntia* genus, native to Mexico [1]. The fruit is ovoid in shape and composed of a thick pericarp (inedible part, known as peel), which accounts for 37–67% of fresh weight, and an endocarp, which accounts for 28–58% of the total fruit in fresh weight [2]. The cactus pear is composed of a considerable quantity of total phenolic acids [2,3], glucose (14%), and insoluble fiber (19.3%), as well as minerals such as potassium and magnesium, all of which, collectively, represent a significant proportion of the recommended dietary intake. These components provide a minimum of 15% of the recommended intake for the Mexican population, thereby establishing the pulp of this fruit as a nutritionally sound option [4]. Additionally, the fruit contains bioactive compounds, including vitamin C, carotenoids, betacyanins, and quercetin, which impart not only organoleptic characteristics, but also antioxidant properties [5].

The pericarp of the cactus pear is typically regarded as a waste product. According to previous studies, the pericarp of the purple prickly pear had a high amount of dietetic fiber (43%), carbohydrates (38.10%), protein (2.78%), lipids (1.75%), and ashes (9.77%) characterized by potassium, magnesium, calcium, and sodium [6]. Moreover, the pericarp contains antioxidants such as phenolic compounds (isorhamnetin, euconmic acid, kaempferol, 3-O-methylquercetin, feruloyl-D-glucose, and piscidic acid) and betacyanins, which are responsible for its reddish–purple color which is associated with beneficial health effects [7,8], as antiviral, anti-inflammatory, and anticancer properties [9]. These nutrients could, potentially, meet dietary recommendations if incorporated into supplements or ingredients of processed foods.

Dietary fiber is a component of the cell wall of plant foods. According to the literature reports, the recommended daily intake of dietary fiber for adult women and men is 25 and 38 g, respectively. This intake is associated with health benefits [10]. An adequate intake of dietary fiber has been demonstrated to exert a beneficial effect on cardiovascular function, with a reduction in the incidence of coronary heart disease observed as a consequence of lowering low-density lipoprotein levels [11]. Dietary fiber plays a role in maintaining good digestive health and adequate intestinal motility, which, in turn, increases stool volume and intestinal transit time [11]. This promotes bowel movement, prevents constipation, provides volume, softens feces, and increases fecal water content [11]. Furthermore, dietary fiber induces a feeling of satiety, which is associated with a lower intake of food and, consequently, energy. This results in the maintenance or reduction of body weight and improvement of health [12]. Accordingly, inadequate dietary fiber intake is a contributing factor to various gastrointestinal diseases. Consequently, individuals often turn to dietary supplements rich in dietary fibers to address these issues and explore their potential therapeutic benefits [13].

Among the most significant supplements endorsed by the American Gastroenterological Association for the management of gastrointestinal disorders are *Psyllium* (*plantago*) and sennosides A and B [14]. The *Psyllium plantago* (*Plantago ovata Forsk*) is a medicinal plant that is notable for its high-fiber content, with a particularly high concentration of soluble fiber (67.20 g/100 g) [15], and other components such as protein, fat, carbohydrates, and bioactive compounds such as phenolics [16,17]. The plant is distinguished by its gelatinous appearance and high viscosity, which is achieved through simple hydration [15]. *Psyllium* is commonly used as a laxative that improves intestinal activity, facilitates digestion, and is used in the treatment of intestinal diseases, including constipation, hemorrhoids, and colon cancer [18]. The clinical use of *Psyllium plantago* in adult women is aimed at maintaining normal glucose levels, increasing satiety, and being useful in the treatment of diabetes, metabolic syndrome, and obesity [16,19].

Other natural stimulant laxatives are the sennosides, which are derived from the pod of *Cassia acutifolia* [20]. The composition of these products is primarily carbohydrates (65.76%) and fiber (10.61 g/100 g) [21]. Some bioactive compounds in the plant of senna are flavonoids, alkaloids, saponins, tannic acid, alkaloids, anthocyanin, quercetin, and coumarins, among others [22]. These agents are employed to address occasional constipation or for bowel emptying. Their laxative effect is observed within a period of 8–12 h post-administration; therefore, they are typically taken before bedtime to allow their effects to manifest the following day [23]. Nevertheless, at the preclinical level, studies have been conducted on albino rats and dogs that have demonstrated that prolonged consumption of the seed for 125 days can result in the dark pigmentation of various organs, including the kidneys, liver, and adrenal gland. This is attributed to the dark pigment present in the seed pericarp; however, this pigmentation is not observed in humans [24]. In general, the concomitant administration of laxatives and diuretics may result in excessive potassium loss. Furthermore, in conjunction with digoxin, it has the potential to exacerbate adverse cardiac effects, necessitating vigilance when co-administered with other pharmaceuticals [23,25]. It is advised that these products should not be used for more than ten days, as they may induce dependence to maintain optimal functioning of the gastrointestinal tract and this must be carried out with a normal diet [20].

The objective of the present study is to determine the physiological and metabolic effects (weight gain, biochemical parameters, and intestinal transit) of the chronic administration of dietary fiber formulations derived from the cactus pear peel (in powder and tablet form) in comparison with commercial products (*Psyllium plantago*, sennosides A and B) in male Wistar rats.

## 2. Materials and Methods

In order to conduct the experimental evaluation, the samples were not encapsulated or compacted into a tablet, as powder was required for the study and administration to the animals. This project was reviewed and approved by the Ethics Committee of the Institute of Health Sciences (ICSa) of the Autonomous University of the State of Hidalgo (UAEH), Mexico (official document CIECUAL/009/2019).

### 2.1. Samples

#### 2.1.1. Cactus Pear Peel Powder

The use of the cactus pear (*Opuntia ficus-indica* L.) was selected for the study, and the fruits were provided by the Mexican Association CoMeNTuna (Consejo Mexicano del Nopal y la Tuna) in Hidalgo, Mexico (latitude of 20°16′12″ N, longitude 98°56′42″ W, and altitude of 2600 m above sea level). The cactus pear fruits were manually harvested during September 2021 and selected in accordance with the Mexican legislation pertaining to non-industrialized food products derived from the *Opuntia* genus. The fruits were of a firm consistency, clean, free of contaminants, and free of damage caused by pests or diseases. Additionally, the fruits exhibited a state of commercial maturity, as determined by the observation of the sinking of the fruit receptacle in accordance with national guidelines [26]. The fruits were divided into three batches, washed, and manually peeled. The mesocarp and pericarp residues were subsequently separated and frozen at −32 °C. Lyophilization was then performed using a VWR26671-581 Labconco (Kansas City, MO, USA) for 96 h at −55 ± 1 °C under a vacuum of 0.040 mbar. The samples were then milled (blender, 38BL52, LBC10, Waring Commercial, Torrington, CT, USA) and sieved to a particle size of <500 μm.

#### 2.1.2. Cactus Pear Peel Tablet Powder

The tablet studied was formulated according to the following specifications: the commercial excipient, i.e., the microcrystalline cellulose (Avicel^®^ PH200 FMC Corp., Spain), constituted 58% of the mixture, which was combined with 40% of cactus pear peel powder. Furthermore, 1% of powder and 1% of magnesium stearate (Panreac-Montplet and Esteban, Barcelona, Spain) was incorporated as a lubricant agent to enhance the quality of the tablets. All components were mixed in a homogenizer at 24 rotations/min (V-mill type MV-6; Turu Grau S.A, Tarrasa, Spain) for 15 min, and, subsequently, compacted using an eccentric tableting machine (Bonnals B-40, Barcelona, Spain). The tablets were subjected to quality testing, such as uniformity of mass, thickness, diameter, hardness, friability, as well as disintegration time [8]. These formulations were established according to preliminary tests in which the excipients were added until complete compaction was achieved. This process has been registered for industrial property registration (MX2018009586A). The registration concerns the formulation of dietary fiber supplements, antioxidants, and excipients as a vehicle for medicaments derived from the epicarp and mesocarp of *Opuntia ficus indica*. In order to contribute to the study, the formulations in powder were used, without reaching the established compaction process.

### 2.2. Dietary Fiber Hydration Properties

The hydration properties (water retention capacity and swelling capacity) of the dietary fiber were evaluated in accordance with the methodology outlined by Meneses et al. [27] and Guillon et al. [28].

#### 2.2.1. Water Retention Capacity (WRC)

The method entailed hydrating a known weight of the sample, subjecting it to a centrifugal force to permit the drainage of the excess supernatant from the pellet, and recording the weight of water retained by the sample.

A sample of 0.5 g of each formulation (P1) was weighed into centrifuge tubes, 10 mL of distilled water was added, and the samples were shaken manually for 10 min. The samples were then left to rest for 24 h at room temperature, and were subsequently subjected to centrifugation in a Beckman Coulter device (model TA 10.250, Woonsocket, RI, USA) at 3000 rpm for 10 min. Subsequently, the supernatant was removed and the sediment (P0) was weighed.

The WRC was calculated in accordance with the following formula:

(1) $$\mathrm{WRC}=\frac{P0-P1}{P1}$$

P1 = initial weight of the sample (g)

P0 = final weight of the sediment (g)

The results are expressed as grams of water retained per gram of sample on a dry basis (g water/g db).

#### 2.2.2. Swelling Capacity (SWC)

The method entailed the dispersion of a known weight of the dry sample in a volume of water in a measuring cylinder, followed by the measurement of the volume occupied by the hydrated fiber after 24 h.

A sample of 2 g of each formulation was weighed in a 25-milliliter graduated cylinder, and the volume occupied by the sample (V1) was subsequently measured. An aliquot of 10 mL of distilled water was then added and shaken manually for a period of 5 min. Subsequently, the samples were left to rest for 24 h at room temperature, after which the final volume of the formulations (V0) was measured.

The swelling capacity was calculated using the following formula:

(2) $$\mathrm{SWC}=\frac{V1-V0\;}{\mathrm{Sample}\;\mathrm{weight}}$$

V1 = initial volume of the formulations (mL)

V0 = final volume of the formulations (mL)

The results are expressed as milliliters of water per gram of sample on a dry basis (mL water/g db).

### 2.3. Animal Preparation

In the preclinical experiment, the behavior of Wistar rats (180–220 g) was observed. The rats were divided into five groups, with six animals in each group, and housed individually in metabolic cages throughout the experiment. The animals were maintained under controlled conditions of constant temperature (22 °C), with a 12:12 h light–dark cycle (lights on at 7:00 h), with the commercial Labdiet^®^ Formulab Diet food brand and water ad libitum, following the experimental procedures outlined in the Mexican Official Norm for Animal Care and Handling (NOM-062-ZOO-1999) [29]. The experiment was conducted over a period of five weeks. During the initial week, the rats underwent an adaptation process involving placement in metabolic cages and daily handling. In the second week, the daily administration of the formulations commenced, with each animal receiving approximately 60 g of food each morning. The metabolic cages were cleaned on a daily basis, and the cages were disinfected and cleaned every 15 days.

### 2.4. Dose Administration

All formulations were administered on a daily basis for a period of four weeks via the intragastric route. A rat of an average weight (200 g) was administered an amount of the fiber sample diluted in 2.5 mL of water, as detailed in Table 1. The calculation of dietary fiber was made based on the amount of fiber indicated on the labels of commercial products: *Psyllium plantago* (17.2 mg/100 g supplement), sennosides A and B (31.28 mg/100 g supplement). The calculation of dietary fiber in cactus pear peel powder was conducted in accordance with the methodology outlined by Manzur-Valdespino et al. [8], resulting in a value of 43 mg/100 g on a dry basis. The cactus peel tablet powder formulation comprised 40% of cactus pear peel (17.2 mg of dietetic fiber) and 58% of microcrystalline cellulose (56.84 mg of dietetic fiber), which is used to compact the tablet. The quantification of fiber present in microcrystalline cellulose (MCC) was calculated theoretically in accordance with the methodology proposed by Ghanbarzadeh et al. [30], with the assumption that 98% of MCC is an insoluble dietary fiber.

Prior to this study, dilution tests were conducted to ascertain the passage of the administration cannula through the gastrointestinal tract of animals and to determine the tolerance for the amount of dietetic fiber, avoiding gastrointestinal symptoms such as diarrhea, constipation, etc.; therefore, the amount of fiber was not the same for all groups.

Additionally, the test aimed to ascertain whether immediate hydration was necessary.

### 2.5. Weight Gain of the Experimental Animals

To determine the extent of weight gain, the weight of the animals was recorded on a daily basis using a digital scale (Adam Equipment AQT-5000, Guadalajara, Mexico). This procedure was conducted in the morning, prior to the administration of the formulations.

### 2.6. Biochemical Parameters

Biochemical profiles were determined by extracting blood from the animals through the tail vein [31], having fasted for a period of eight hours. The concentrations of total cholesterol (TC), high-density lipoprotein (HDL), and triglycerides (TG) in the blood were determined using commercially available kits (Spinreact^®^, Spinreact S.A.U., Girona, Spain) in accordance with the instructions provided by the manufacturers.

In order to interpret the values, the parameters for Wistar rats established by Sari [32] and Boehm [33] were utilized as a reference. The results are expressed in milligrams per deciliter (mg/dL).

### 2.7. Intestinal Transit

#### 2.7.1. Moisture of Feces

The moisture content of the fecal matter was monitored at regular intervals throughout the course of the experiment. This was accomplished by collecting and evaluating the fecal matter on a weekly basis, with samples obtained every two days. The moisture content was determined by measuring the weight of the feces before and after drying. To ascertain the moisture content, a quantify of one gram of feces was collected in preformed aluminum containers. The percentage of moisture was calculated by subtracting the initial weight of the fecal granules from the dry weight after drying in an oven at 105 °C for 90 min [34].

#### 2.7.2. Ratio of Intestinal Transit Time

The relationship between intestinal transit time and other variables was evaluated using the activated charcoal test. On the 28th day of the experiment, the rats were administered with the treatments. Subsequently, 2 mL of a 5% charcoal suspension with 5% arabic gum was administered via a flexible intragastric tube 30 min later.

The animals were sacrificed 30 min after the administration of activated charcoal and arabic gum. Subsequently, the stomach and small intestine were excised, and the length of the intestine was measured (from the pyloric sphincter to the ileocecal junction) using a metric tape measure. Additionally, the distance traversed by the colored food bolus or by the activated charcoal was documented. The degree of intestinal motility was quantified by calculating the percentage of intestinal transit, defined as the distance traversed by the activated charcoal divided by the total length of the small intestine of the evaluated rat, multiplied by 100 [35].

(3) $$T\;\left(\%\right)=\frac{B}{A}\times 100$$

T: intestinal motility

B: distance traveled by activated carbon (cm)

A: total length of the small intestine (cm)

### 2.8. Euthanasia

Upon the conclusion of the study, all animals were humanely euthanized in accordance with the guidelines set forth in NOM 062-ZOO-1999 [29] by the cervical dislocation method, which was performed swiftly and without distress to the animals. The animals were then placed in corresponding RPBI bags.

### 2.9. Statistical Analysis

The results are presented as the mean ± standard error of six animals per group to evaluate the effects of the formulation administration. The unpaired student’s *t*-test was used to compare the effects of the fiber sample and its control. The homogeneity of variances was assessed prior to a two-way analysis of variance (ANOVA), followed by a Tukey’s post hoc test to compare the differences of different treatments. The significant differences were considered to be those with a *p*-value lower than 0.05.

## 3. Results and Discussion

The ensuing discussion and analysis pertain to the hydration properties of the two cactus pear peel formulations and the two commercial supplements. The water retention capacity (WRC) and the swelling capacity (SWC) were evaluated as a means of assessing these properties. Moreover, the impact of these formulations was evaluated in male Wistar rats to assess changes in weight gain, in the biochemical parameters, and intestinal transit.

### 3.1. Fiber Hydration Properties in the Samples

The hydration properties of the fiber are contingent upon the physical characteristics of the vegetable foods and a correlation could be drawn between these properties and physiological effects [36]. The water retention capacity (WRC) is a property that expresses the maximum amount of water (in milliliters) that can be retained per gram of dry matter in the presence of an excess of water. Moreover, it influences the viscosity of materials, including foodstuffs, which can either facilitate or impede processing [37].

Table 2 presents the results of the hydration properties of dietary fiber. The *Psyllium plantago* sample exhibited a high water retention capacity (WRC) of 17.23 g water/g db, which can be attributed to its composition and high concentration of fiber, predominantly soluble fiber [22,38]. The remaining samples demonstrated comparatively lower WRC values (2.35 and 3.95 g/g db), while the tablet displayed a WRC of 2.35 g/g db. The WRC values were found to be higher or similar to those observed in other samples with a high content of insoluble fiber, including apple, grapefruit, orange, and lemon waste with values between 1.62 and 2.26 g/g db [39]. As a result, these samples demonstrated an optimal dispersion of water which could be related to the formation of viscous gels. These properties could cause the same properties within the gastrointestinal tract, resulting in a reduction in glucose absorption and the generation of a lower glycemic index. Additionally, these fibers might afford other benefits for the maintenance of the intestinal microbiota [37].

The swelling capacity (SWC) is defined as the volume occupied by a fiber following hydration under specific conditions [40]. In other words, water moves toward the solid structure of the cellular matrix, spreading its macromolecules until the generation of the swelling action [41]. All groups exhibited a range of 3.38–4.38 mL/g, which is comparable to the findings from fruit residues (cooked apple, pear, and dried date) (3.9–7 mL/g db) [42], oat bran (2.3 mL/g db), and apple fiber (3.4 mL/g db) [43]. The SWC is a property that can be related to effects on gastric emptying, which is slowed down, leading, as a result, to an increase in stomach volume and a sensation of fullness and satiety [44]. Furthermore, it is associated with an increase in fecal volume [45].

The physical properties of dietary fiber, including particle size, porosity, cell wall components (mainly polysaccharides), and interconnectivity, influence the ability to retain water [46]. It is of paramount importance to acknowledge that the primary components of the cactus pear peel fiber samples are cellulose and hemicellulose (insoluble fiber), which exhibit minimal swelling [47].

### 3.2. Weight Gain of the Experimental Animals

**Figure 1:**
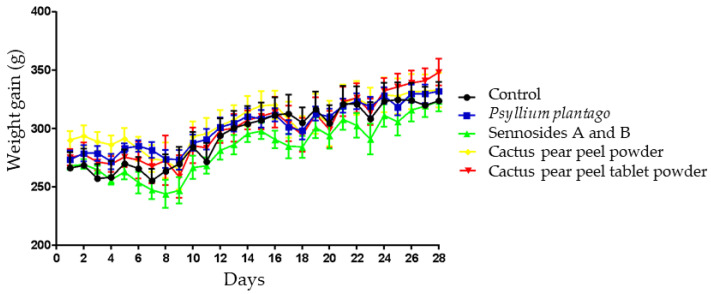
Weight gain (g) of the experimental groups after administration for 28 days. Each point represents the mean ± standard error of the mean (SEM), *n* = 6.

Figure 1 illustrates the change in body weight gain expressed in grams for the experimental animals during the course of the treatment. The animals that received the different formulations were maintained at a healthy weight according to the age of this strain of rat (according to Cossio-Bolaños [48]), with no significant differences (*p* < 0.05) observed between the control and the different experimental groups during the treatment period of 28 days. This result aligns with the findings of another study, which demonstrated that the administration of bean peel husk to healthy rats for a three-week period led to normal growth and to the promotion of gut health through the oligosaccharides present in the diet [49]. The aforementioned study was conducted on healthy rats, indicating that the administration of fiber does not affect the normal development of the animals. Nevertheless, the administration of fiber to animals with induced obesity resulted in a reduction in weight gain [50].

In contrast, Delzenne et al. [51] demonstrated that a brief period of fiber intake has a negligible impact on body weight, with physical activity and the consumption of low-energy foods exerting a more pronounced effect [52].

### 3.3. Biochemical Profile

Table 3 demonstrates the influence of the administration of the formulations, comprising *Psyllium plantago*, sennosides A and B, cactus pear peel powder, cactus pear peel tablet powder, and the control group (water), on the biochemical values of the rats over the 28 days of the treatment period, with evaluations conducted on day 0 and day 28.

On day 0, the glucose level of the Wistar rats was 127.85 mg/dL. The groups administered with *Psyllium* and cactus pear tablets demonstrated a reduction in their glucose level values at the conclusion of the treatment period. This could be due to the fact that, when these fibers come into contact with water, they form a gelatinous and viscous mass which prevents the absorption of glucose through the inhibition of the enzymatic activity [53].

Conversely, according to the initial values present in the groups on day 0 (72.30 mg/d), the groups sennosides A and B and cactus pear peel tablets exhibited a slight increase at the end of the treatment, having a similar behavior to that of the control group. This may be attributed to the metabolic processes of the animals. The remaining samples did not exhibit any notable differences throughout the course of the treatment period. These findings are comparable to those observed in three fruit by-products (apple residue, orange bagasse, and passion fruit peel) when used as supplements in Wistar rats for 34 days, maintaining normal values (80 to 94 mg/dL) for these animals [54].

Dietary fiber plays a role in the absorption of total lipids and the reduction in the activity of pancreatic enzymes. This occurs through entrapment in viscous networks such as those formed by *Psyllium plantago* [55], and the elimination of bile acids through feces facilitates the digestion and absorption of dietary lipids. Therefore, lipids coated with fiber are not reabsorbed in the ileum (enterohepatic circulation), resulting in a reduced amount of bile acids entering the liver. This, in turn, leads to a decrease in blood cholesterol synthesis and, consequently, a reduction in lipid levels within the organism [40,46]. In this study, we employed cactus pear peel powder, which may exhibit an effect analogous to that of recognized fibers, such as *Psyllium*, with the objective of reducing blood glucose levels or maintaining them within normal ranges for blood lipids.

With regard to the cholesterol parameter, the initial value in the rats was 83.30 mg/dL. By day 28, all samples exhibited a statistically significant decrease in cholesterol levels in comparison with the initial values, which ranged between 62 and 85 mg/dL. However, the *Psyllium plantago* sample remained unaltered throughout the course of the treatment, while the cactus pear peel tablet powder was the sole sample to exhibit a higher value than the initial (105.53 mg/dL), exceeding the reference values.

The ingestion of foods with elevated fiber content has the potential to diminish serum cholesterol levels, contingent on their solubility and viscosity [56]. The literature indicates that dietary fiber supplementation over an extended period and in larger quantities than those in the present study can result in significant changes in total cholesterol levels [57]. As stated by Guo et al. [58], the efficacy of dietary fiber (DF) in reducing lipid levels is contingent upon its particle size and soluble fraction content, with varying degrees of effectiveness. The soluble fraction content determines the physicochemical properties and bioactivities of the fiber.

The modified microcrystalline cellulose (used in the cactus pear peel tablet powder) has been employed in a variety of applications within the pharmaceutical and food industries. The modified cellulose is characterized by the defining characteristics of the modified cellulose. Once the hydrogen bonds are disrupted and the crystallinity is lost, the cellulose derivative becomes water-soluble. A number of studies have indicated that the gelation property of dietary fiber may result in a reduction in bile acid absorption. This is thought to be due to an increase in intestinal viscosity and a higher bile acid excretion, which, in turn, causes an increase in hepatic cholesterol synthesis as a result of the higher acid excretion [59,60,61]. Therefore, this could explain the increase in cholesterol in the group that consumed the cactus pear tablet.

High-density lipoproteins (HDL) in the blood are responsible for transporting cholesterol to the liver for subsequent metabolism and final transport to the intestine and elimination in the lower gastrointestinal tract [62]. The baseline value for the animal population under study was 27.57 mg/dL at the beginning of the study and it was maintained until day 28 in all rats administered with the fiber formulations with the exception of *Psyllium plantago*, which led to a notable decline. These findings are consistent with those reported by Olagunju and Omoba [63], evaluate functional products with high-fiber content, and establish that HDL parameters remained within normal ranges during the experimental period. The presence of bioactive compounds, mainly phenols, has been referenced in the samples evaluated, and these compounds had the potential effect of antioxidants increasing HDL or remaining stable, a phenomenon which is correlated with a lower risk of atherosclerotic diseases [64].

### 3.4. Intestinal Transit

#### 3.4.1. Moisture of Feces

**Figure 2:**
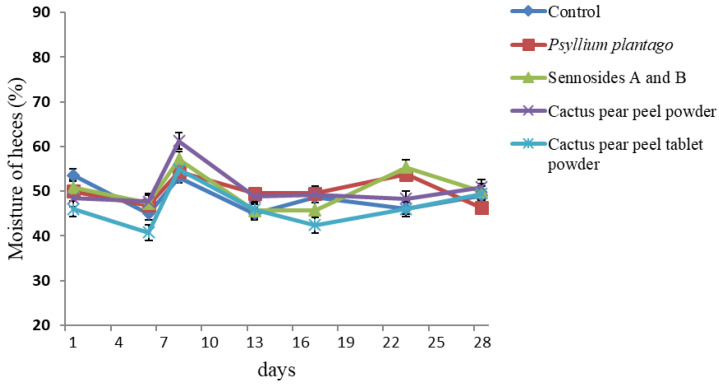
Moisture of feces of the five experimental groups expressed in percentage during the 4 weeks of supplementation (28 days), *n* = 6.

Figure 2 depicts the fluctuations in fecal moisture levels across the five experimental groups over a four-week span. In general, the samples exhibited maintained levels between 40 and 60%, with no statistically significant differences observed between the samples and the control. These results are higher than those reported by Macagnan et al. [54], who evaluated the fecal moisture of rats administered with three fruit by-products (apple residue, orange bagasse, and passion fruit peel) and found percentages ranging between 32.84 and 39.88%. Insoluble dietary fiber resists digestion in the upper intestine and reaches the colon as structurally intact molecules, which are used to form stool. In a study where bay leaves were administered to rats for 28 days, a positive effect was observed, with an increase in the percentage of fecal moisture (between 40 and 60%) due to the action of the insoluble fiber [65].

It can be reasonably deduced that the hydration properties of dietary fiber play a significant role in regulating adequate gastric emptying within the digestive tract and, thus, cause a crucial physiological effect [66]. Insoluble fiber is more resistant to fermentation by bacteria in the colon. Additionally, it has the ability to passively attract water, which promotes volume and the softening of the feces, as well as relaxation (peristalsis) of the large intestine [67].

#### 3.4.2. Gastrointestinal Transit Time

**Figure 3:**
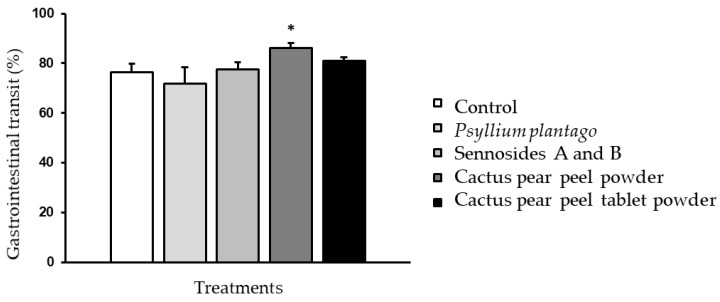
Gastrointestinal transit (%) of the control group and the groups administered with *Psyllium plantago*, sennosides A and B, cactus pear peel powder, and cactus pear peel tablet powder at the end of the treatment (28 days). Each point represents the mean ± standard error of the mean (SEM), *n* = 6. * *p* < 0.05.

Figure 3 shows the results of the measurement in centimeters traveled by the bolus, expressed as the percentage of gastrointestinal transit in relation to the entire small intestine.

Figure 3 illustrates that the cactus pear peel powder exhibited a significantly higher percentage (86.19%) compared to the other samples. The cactus pear peel tablet powder exhibited a value of 80.84%, while the sennosides A and B, as well as *Psyllium plantago*, demonstrated lower values (77.55% and 71.88%, respectively). However, these values were comparable to those observed in the control group (76.27%). The results of the fiber samples were comparable to the values of another study in which the administration of date pulp extract resulted in an acceleration of intestinal transit time (81.02%) in comparison to the control group (68.46%) and the palm sap group (74.01%). This acceleration could be attributed to the insoluble dietary fiber present [68]. The cactus pear peel formulations exhibited a greater distance traveled in less time, indicating an acceleration in intestinal transit due to the high content of insoluble fiber [68]. The result can be attributed to the inability of insoluble fiber to dissolve in water, a phenomenon which results in accelerated gastrointestinal transit time and a reduction in the risk of constipation. This, in turn, serves to protect the colon by reducing prolonged exposure to cytotoxic substances, which are known to be harmful to human health [69,70].

In brief, powders are more prone to segregation issues and may present challenges in ensuring uniform distribution of the active pharmaceutical ingredient [71]. Conversely, tablets are compressed solid dosage forms more accessible for oral use, and they provide precise dosing and stability of the active compounds. In addition, the ingredient added to the tablets, i.e., crystalline microcellulose, has very specific functions to achieve a good compaction, but it also adds insoluble fiber; its effect on the determined parameters in this study were not evaluated.

## 4. Conclusions

The findings indicate that the administration of fiber derived from the prickly pear peel in various formulations (powder and tablets) over an extended period can enhance physiological and metabolic parameters, including a reduction in serum levels of glucose, triglyceride levels, and gastrointestinal transit time in healthy rats.

These findings may have a beneficial impact at the clinical level for phytopharmaceuticals that enhance the efficacy of drugs with metabolic properties. Nevertheless, this study is not without limitations. These include the characteristics of the study such as the number of samples per group, the variation in fiber between the formulations, and the effect on the healthy population, all of which, collectively, limit the ability to draw more conclusive results. Further research should include the investigation of a placebo group or a group that receives only the excipient of the formulations, the testing of different doses of fiber, and the determination of the effect on various different pathologies with metabolic alterations, such as diabetes, constipation, and obesity, among others.

## 5. Patents

Industrial property registration number MX2018009586A—Formulation of dietary fiber supplement, antioxidants, and excipient as a vehicle of medicaments from epicarp and mesocarp of opuntia ficus indica.

## Figures and Tables

**Table 1:** Formulations and quantities administered per group of animals.

Cluster	Treatment	Administered Amount	Fiber Content (mg)
1	Control	2.5 mL water	-
2	*Psyllium plantago*	150 mg + 2.5 mL water	30.49
3	Sennosides A and B	97.5 mg + 2.5 mL water	25.80
4	Cactus pear peel powder	182.5 mg + 2.5 mL water	74.04
5	Cactus pear peel tablet powder	182.5 mg + 2.5 mL water	135.12

**Table 2:** Fiber hydration properties of the samples.

Formulation	Water Retention Capacity (WRC) (g water/g db)	Swelling Capacity (SWC) (mL water/g db)
*Psyllium plantago*	17.23 ± 0.47 ^d^	4.38 ± 0.18 ^a^
Sennosides A and B	3.95 ± 0.01 ^b^	4.13 ± 0.53 ^a^
Cactus pear peel powder	5.33 ± 0.64 ^c^	3.38 ± 0.18 ^a^
Cactus pear peel tablet powder	2.35 ± 0.24 ^a^	3.50 ± 0.00 ^a^

^a,b,c,d^ Values with different lowercase letters in the same column indicate a significant difference between the treatments, *p* < 0.05.

**Table 3:** Biochemical values in Wistar rats during 28 days of treatment (mg/dL).

Normal Parameters (mg/dL) *	Treatments	Day 0 **	Day 28
Glucose (91–166)	Control	127.85 ± 5.37 ^Aa^	124.44 ± 12.51 ^Ab^
	*Psyllium plantago*	127.85 ± 5.37 ^Ba^	96.96 ± 3.26 ^Aab^
	Sennosides A and B	127.85 ± 5.37 ^Aa^	116.42 ± 16.83 ^Ab^
	Cactus pear peel powder	127.85 ± 5.37 ^Aa^	116.06 ± 19.77 ^Ab^
	Cactus pear peel tablet powder	127.85 ± 5.37 ^Ba^	68.30 ± 12.48 ^Aa^
Triglycerides (35–186)	Control	72.30 ± 2.84 ^Aa^	123.75 ± 10.40 ^Bc^
	*Psyllium plantago*	72.30 ± 2.84 ^Aa^	74.44 ± 15.27 ^Aab^
	Sennosides A and B	72.30 ± 2.84 ^Aa^	115.19 ± 22.55 ^Bbc^
	Cactus pear peel powder	72.30 ± 2.84 ^Aa^	49.52 ± 18.33 ^Aa^
	Cactus pear peel tablet powder	72.30 ± 2.84 ^Aa^	110.95 ± 10.03 ^Bbc^
Cholesterol (42–77)	Control	83.30 ± 2.77 ^Ba^	62.82 ± 6.59 ^Aa^
	*Psyllium plantago*	83.30 ± 2.77 ^Aa^	85.19 ± 11.34 ^Ab^
	Sennosides A and B	83.30 ± 2.77 ^Ba^	69.96 ± 2.61 ^Aab^
	Cactus pear peel powder	83.30 ± 2.77 ^Ba^	63.84 ± 2.25 ^Aa^
	Cactus pear peel tablet powder	83.30 ± 2.77 ^Aa^	105.53 ± 9.31 ^Bc^
HDL (20–42)	Control	27.57 ± 4.20 ^Aa^	30.26 ± 9.24 ^Aa^
	*Psyllium plantago*	27.57 ± 4.20 ^Ba^	10.76 ± 5.07 ^Aa^
	Sennosides A and B	27.57 ± 4.20 ^Aa^	28.91 ± 9.95 ^Aa^
	Cactus pear peel powder	27.57 ± 4.20 ^Aa^	22.19 ± 10.67 ^Aa^
	Cactus pear peel tablet powder	27.57 ± 4.20 ^Aa^	18.83 ± 6.48 ^Aa^

^A,B^ Values with different capital letters on the same line indicate a significant difference between the time of each treatment (0 and 28 days), *p* <0.05. ^a,b,c^ Values with different lowercase letters in the same column indicate a significant difference between the same treatments, *p* < 0.05. * Source [32,33]. ** The values in day 0 correspond to the average of the biochemical parameters of a group of healthy animals (*n* = 6) at the beginning of the experiment.

## Data Availability

The original contributions presented in the study are included in the article, further inquiries can be directed to the corresponding author.
